# Increasing the sensitivity of reverse phase protein arrays by antibody-mediated signal amplification

**DOI:** 10.1186/1477-5956-8-36

**Published:** 2010-06-22

**Authors:** Jan C Brase, Heiko Mannsperger, Holger Fröhlich, Stephan Gade, Christian Schmidt, Stefan Wiemann, Tim Beissbarth, Thorsten Schlomm, Holger Sültmann, Ulrike Korf

**Affiliations:** 1 Division of Molecular Genome Analysis, German Cancer Research Center, Heidelberg, Germany; 2 Department of Medical Statistics, University Medicine Göttingen, Göttingen, Germany; 3 Martini-Clinic, Prostate Cancer Center, University Medical Center Hamburg-Eppendorf, Hamburg, Germany

## Abstract

**Background:**

Reverse phase protein arrays (RPPA) emerged as a useful experimental platform to analyze biological samples in a high-throughput format. Different signal detection methods have been described to generate a quantitative readout on RPPA including the use of fluorescently labeled antibodies. Increasing the sensitivity of RPPA approaches is important since many signaling proteins or posttranslational modifications are present at a low level.

**Results:**

A new antibody-mediated signal amplification (AMSA) strategy relying on sequential incubation steps with fluorescently-labeled secondary antibodies reactive against each other is introduced here. The signal quantification is performed in the near-infrared range. The RPPA-based analysis of 14 endogenous proteins in seven different cell lines demonstrated a strong correlation (r = 0.89) between AMSA and standard NIR detection. Probing serial dilutions of human cancer cell lines with different primary antibodies demonstrated that the new amplification approach improved the limit of detection especially for low abundant target proteins.

**Conclusions:**

Antibody-mediated signal amplification is a convenient and cost-effective approach for the robust and specific quantification of low abundant proteins on RPPAs. Contrasting other amplification approaches it allows target protein detection over a large linear range.

## Background

In recent years reverse phase protein arrays (RPPA) have proven themselves as useful experimental platform for the validation of biomarker candidate proteins in biological and clinical samples [1-6]. RPPA are considerably faster than conventional techniques such as mass spectrometry, western blotting, 2-D PAGE, and allow the analysis of hundreds of samples in parallel. In addition, measurements can be made with high accuracy and reproducibility. The basic idea of RPPA implies that all samples are spotted in parallel on solid-phase carriers. Samples can be printed either as serial dilutions or in a single concentration but as multiple replicate spots [7]. The detection of a specific protein or a certain phosphorylation site is carried out with a single and highly specific antibody per slide, and the fraction of captured antibodies is mostly visualized with secondary antibodies. Recently, near infrared fluorescence-based detection was reported as useful for reverse phase protein microarrays [8,9]. Routine applications involved analyzing the activation status of signaling pathways [10], protein profiling after RNAi-based silencing experiments [11], as well as of tumor biopsies [12,13].

Critical for the outcome of RPPA-based measurements are first of all the sensitivity and specificity of primary antibodies but also of the signal detection method since samples are delivered as tiny droplets on solid phase carriers and no further separation steps are possible. In addition, proteins of interest may be expressed at a low level and their visualization requires means for signal amplification. Tyramide signal amplification (TSA) has been widely applied to increase the sensitivity of RPPAs [14-18]. However, TSA linked with a streptavidin-biotin strategy can produce unspecific signals that may interfere with the signal of interest [19].

**Figure 1:**
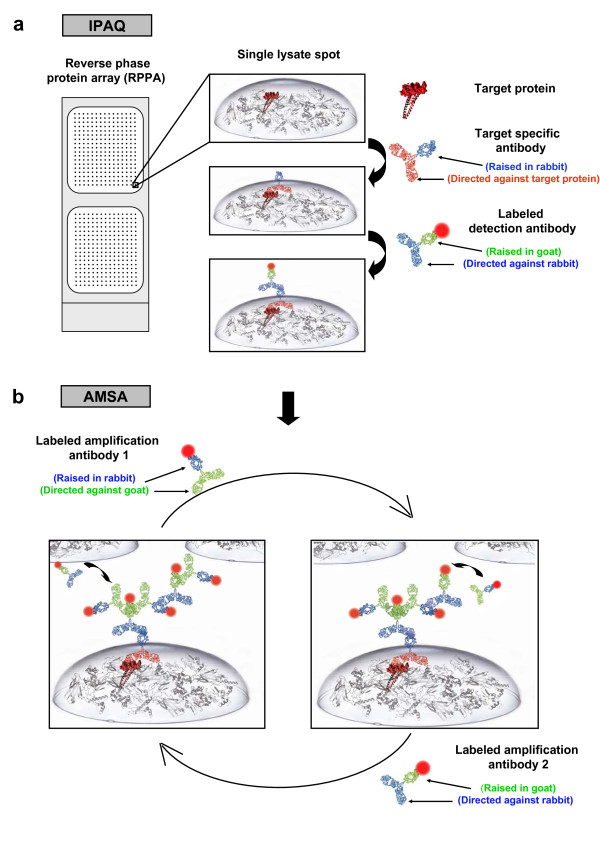
**Overview antibody-mediated signal amplification**. (a) Samples are dispensed using a microarrayer, target proteins are detected with highly specific antibodies and visualized by incubation with near-infrared dye-labeled secondary antibodies. Fast-Green FCF based normalization defines a spot-specific normalization factor. (b) Using the antibody-mediated signal amplification assay (AMSA) the antibody-based target protein detection step is followed by additional cycles of incubating with two different NIR-labeled species-specific secondary antibodies to increase the density of fluorescent signals. Both secondary antibodies were selected to recognize the respective other species.

The fluorescence-based signal amplification method introduced here avoids the use of the streptavidin-biotin system and achieves signal amplification by using fluorescently labeled antibodies from two different species selected to recognize the respective other species. With increasing numbers of alternating incubation cycles the intensity of fluorescent signals was stronger and a highly sensitive quantitative read-out was obtained (Figure 1). This approach was named antibody-mediated signal amplification (AMSA). Working steps were adapted to a fully automated procedure. AMSA was demonstrated to be a robust and specific approach to increase the sensitivity of the recently reported IPAQ strategy [8]. Using a spike-in experiment with recombinant JNK1-protein the lower limit of detection (LOD) was reduced almost 10-fold compared to standard near infrared fluorescence readout using the IPAQ strategy. Additional experiments demonstrated that AMSA is especially useful to improve the linear range of signal detection for proteins expressed at a low level.

## Methods

### Cell culture conditions and cell lysis

Human colon cancer cell lines HCT15 (CCL-225), HCT116 (CCL-247), H508 (CCL-253), SW620 (CCL-227), HT29 (HTB-38), RKO (CLR-2577), and SW480 (CCL-228) as well as the breast cancer cell lines HCC1954 (CRL-2338), MDA-MB-231 (HTB-26), BT-474 (HTB-20), SK-BR-3 (HTB-30), MCF-7 (HTB-22), and T47D (HTB-133) were obtained from American Type Culture Collection (Manassas, USA). Breast cancer cell lines were cultivated under conditions recommended by ATCC (ATCC serum and medium annotation, http://www.lgcpromochem-atcc.com). All colon cancer cell lines were cultivated in RPMI 1640 medium (ATCC) containing 10% FCS and 1% Pen/Strep (both Invitrogen, Karlsruhe, Germany). Cell lines were grown until 90% confluence and split 3 times *per *week. For lysis, cultured cells were incubated with trypsin (Sigma, Munich, Germany) and lysed in 1 ml M-PER (Pierce, Rockford, USA) containing 2 mM ortho-sodium-vanadate (Sigma), 10 mM NaF, and Complete mini protease inhibitor. The protein concentration was determined in duplicate by BCA Protein Assay (Pierce). Protein lysates were stored at -80°C until use.

### Tissue preparation

Prostate tissue samples were collected at Martini-Clinic, Prostate Cancer Center (University Medical Center Hamburg-Eppendorf, Germany). Samples were acquired during radical prostatectomy and sectioned with a microtome. One slice of prostate tumor tissue was processed per experiment. Mouse liver tissue and collected prostate tissue were lysed with modified T-PER buffer (Pierce). 10 μl of lysis buffer were used per mg of tissue sample. Samples were homogenized 4 minutes with a tissuelyser (Qiagen, Hilden, Germany). Cell debris was subsequently pelleted (12 minutes, 13000 rpm) and the supernatant was passed through a Qiashredder tube (Qiagen).

### Printing of protein microarrays

Protein lysates and spike-in experiments with recombinant His-tagged JNK (Invitrogen) were serially diluted with protein lysis buffer. To prepare samples for inkjet-spotting using the Sprint (ArrayJet, Roslin, Scotland) an equal volume of spotting-buffer (50% (v/v) Glycerol and 0.05% (w/v) Triton × 100 in ddH_2_0) was added to each sample resulting in a final glycerol concentration of 25%.

A pin tool spotter, the 2470 Arrayer (Aushon, Billerica, MA, USA), was employed to print serial dilutions of cell line lysates (SW480, HCT15, HCT116, SW620, HT29, HCC1954, MDA-MB-231, BT-474, SK-BR-3, MCF-7 and T47D). Dilution series comprised 14 steps and were produced by diluting with lysis buffer by 33% per step. Samples were printed directly after adding Tween20 to a final concentration of 0.05% (v/v).

Samples were always printed onto nitrocellulose coated glass-slides (Grace Biolabs, Bent, OR, USA). Each spot corresponded to a final drop volume of 0.3 nl (pin tool spotter) or 0.6 nl (non-contact inkjet printer). The spot-to-spot distance was set to 320 μm and the spot diameter was 140 μm. Slides were stored at 4°C and used within a week.

### Near infrared target protein detection on microarrays

Slides were blocked with a mixture of 33% Odyssey blocking buffer (LI-COR, Lincoln, USA), 1% BSA, and 0,02% NP40 in PBS overnight at 4°C. Primary antibodies were diluted in a buffer with background-reducing components (Dako, Glostrup, Denmark). Slides were incubated for 2 h with primary antibodies and subsequently immersed in wash buffer (1x PBS, 0.02% NP40 and 0.02% SDS) four times for 5 minutes. Next, slides were incubated with Alexa680-conjugated secondary antibody (Invitrogen, dilution 1:8,000) for 30 minutes. Washing was performed as described above. All washing and incubation steps were carried out at RT with gentle shaking. Finally, slides were rinsed in water and air-dried at room temperature.

The following detection antibodies were employed for western blotting and RPPA-based analysis: JNK (BD Biosciences, San Jose, USA), CDK4, Cyclin D1, GSK, PP2AA, PP2AB, PDK1, beta-Catenin, RB, Smad 2/3, SRC (Cell Signaling Technologies, Danvers, USA), ERK 1/2, NF-κB, PCNA, Stat3 (Santa Cruz, Santa Cruz, USA), KLK3 (Sigma), JNK, PKC, PLCγ (Abcam, Cambridge, USA), and pERK (R&D, Minneapolis, USA).

### Antibody-mediated signal amplification on microarrays

Incubation with primary antibodies was carried out as described in the previous section. All working steps for antibody-mediated signal amplification were integrated into an automated procedure. A robotics protocol was established to increase and simplify the throughput. The 96-channel head robot Biomek FXP (Beckmann Coulter, Harbor Boulevard, USA) was used for all assay steps thus reducing hands-on time and minimizing experimental variation.

Anti-rabbit Alexa680-labeled (raised in goat) and anti-goat Alexa680-labeled (raised in rabbit) antibodies (both Invitrogen) were applied consecutively in a total of four cycles (dilution 1:8,000). Anti-mouse Alexa680 labeled (raised in goat) antibody was used in the first cycle for the detection of primary antibodies raised in mouse. Secondary antibodies employed for signal amplification were derived from commercial sources and of highest purity. Slides were washed four times for 5 minutes between automated incubation steps. Each secondary antibody was incubated for 30 minutes.

### Western Blot detection

SDS-Page and Western immunoblotting analyses were performed using 5 to 20 μg protein lysate. Standard near infrared detection was applied as described elsewhere [20]. The protocol for antibody-mediated signal amplification was adapted to a larger volume. Tyramide signal amplification was carried out in parallel to compare directly between both amplification methods. TSA Kit #11 (Invitrogen) was employed according to the manufacturer's instructions. Endogenous peroxidase was treated with 3% H_2_O_2 _for one hour. A 1:250 dilution of the HRP conjugated secondary antibody was applied. Peroxidase catalyzed signal amplification was carried out for 10 minutes and Streptavidin-Alexa680 (Invitrogen) was incubated for 20 minutes.

### Statistical analysis

Slides were scanned with the Odyssey NIR scanner (LI-COR). Image analysis was carried out with GenePix-Pro 5.1 (Axon Instruments, Sunnyvale, USA). Spot intensity was corrected for background and noise due to unspecific antibody binding. Mean and SD were calculated for all replicates. Data sets were analyzed within the statistical computing environment R.

The detection limit was determined as follows: First, the signal intensity mean of a certain dilution step has to be greater than the sum of 2 x standard deviation (SD) of S_0 _and the mean of S_0 _with S_0 _corresponding to the lowest concentration of a dilution series. Thus, S_0 _was considered as background signal [21] ensuring a significant difference of signals from background. Second, in a dilution series signals must increase continuously with increasing proteins concentrations. The lowest concentration of a certain serial dilution complying with both requirements is defined as limit of detection (LOD). To sum up, the detection limit corresponded to the smallest concentration of a certain serial dilution that can be distinguished from the experimental background as well as from the next higher concentration. A detection limit is a specific measure for a specific detection antibody, detection method, and a specific sample. The curve fitting model and the normalization approach are described in detail in Additional file 1.

## Results

### Optimizing AMSA to a robust and specific amplification method

Important experimental parameters such as blocking and wash buffer composition, primary and secondary antibody concentration, and the effect of background reducing agents were analyzed systematically to establish a new amplification approach for RPPA. For example, a spike-in approach with recombinant JNK1 protein was employed to assess the detection limit of the new amplification method. Beforehand, the specificity of all antibodies used in the amplifications steps was confirmed by probing suitable controls printed on microarrays such as human immunoglobulins and recombinant JNK protein. Signal detection omitting the target specific anti-JNK1 antibody yielded no signals (Additional file 1, figure 1a). Data also demonstrated that species-specific antibodies used as part of the amplification procedure did not cross react with human immunoglobulins potentially present in clinical samples of human origin. On the other hand, the presence of human immunoglobulins was confirmed by probing a replicate slide with anti-human IgG antibody (Additional file 1, figure 1b), and the specificity of the JNK1-detection was demonstrated as well (Additional file 1, figure 1c).

**Figure 2:**
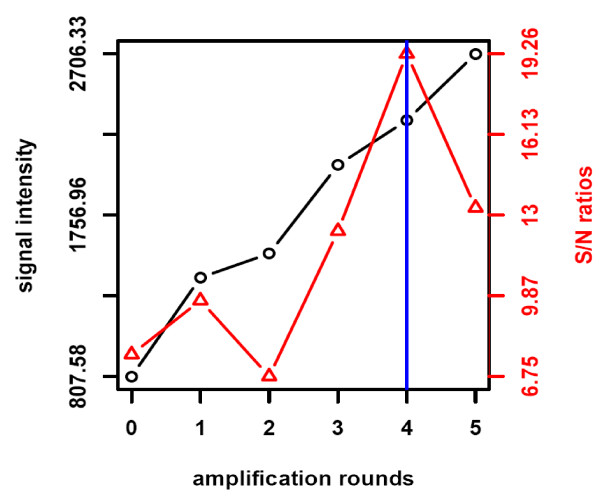
**Optimizing number of amplification cycles**. Twelve replicate spots of a colon cancer cell line containing 35 fg pERK were printed on nitrocellulose-coated slides. Detection of pERK was performed by standard near-infrared detection and AMSA comprising different rounds of amplification. Median fluorescence (black) and signal-to-noise ratios (red) were calculated. The blue line summarizes the results for AMSA optimization.

As a side-effect of signal amplification an increase of background signal was supposed. Therefore, the AMSA-dependent background signal gain was quantified using a spike-in of phosphorylated recombinant ERK1. This way, the best trade-off between specific and background signal intensity gain was assessed. RPPA signal detection was based on standard NIR or AMSA with one to five rounds of signal amplification (Figure 2). Thus, background signal intensity gain correlated with the number of amplification steps performed. After the first two cycles of amplification signal-to-noise ratios were not improved compared to those obtained by standard NIR detection. However, after three amplification cycles the signal-to-noise ratio started to increase and the best result was obtained by carrying out four amplification cycles and this number of cycles was consequently chosen as basis for the AMSA procedure.

**Figure 3:**
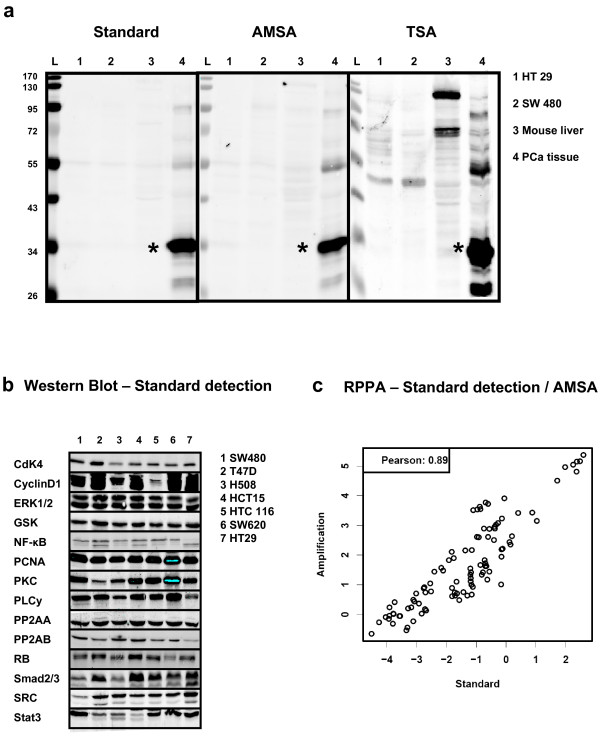
**Comparing standard NIR detection and AMSA**. (a) Western blot specificity of TSA and AMSA. Detection of PSA (star-symbol) using standard NIR procedure (scan intensity 5), antibody-mediated signal amplification (scan intensity 2.5) and TSA (scan intensity 2.5). Each lane corresponds to loading 5 μg total protein from prostate cancer tissue or PSA-free negative controls: Colon cancer cell lines HT29 (1), SW480 (2), mouse liver lysate (3), and prostate cancer tissue (4). (b) Western blot analysis of 14 proteins in seven human cancer cell lines with standard NIR detection. (c) Samples were analyzed by RPPA to compare standard NIR detection and AMSA using the same set of antibodies as shown for the Western blot. The correlation analyses were based on log signal intensities.

The specificity of the AMSA approach was validated by detecting PSA in different samples on Western blots (Figure 3a) and compared to blots detected in parallel by standard NIR and TSA-based signal amplification. In detail, the presence of PSA was probed in a human prostate cancer lysate and in samples serving as PSA-free negative controls such as human colon cancer cell lines (HT29, SW480) or homogenized mouse liver. Strong and specific PSA signals were detected only in prostate cancer samples and when using standard NIR detection and AMSA. The TSA detection yielded also a strong PSA band on Western Blots. Nonetheless, several additional bands were observerd in prostate tumor samples as well as for the mouse liver lysates. To sum up, unspecific bands were not observed in cell line samples and when using standard NIR detection or AMSA. Thus, the numerous additional bands emerging during the TSA procedure were also observed when the anti-PSA antibody was omitted (data not shown) and possibly resulted from insufficient masking of biotin proteins as artifact of the TSA detection.

Aiming at a more comprehensive validation for a larger set of target proteins 14 highly expressed signaling proteins (CDK4, CyclinD1, ERK1/2, GSK, NF-κB, PCNA, PKC, PLCy, PP2AA, PP2AB, RB, SMAD2/3, SRC, STAT3) were chosen. Western blot analysis confirmed that all proteins are indeed expressed in the human cancer cell lines (Figure 3b). The samples were also printed on nitrocellulose-coated slides and probed with the same set of primary antibodies to compare standard NIR detection with antibody-mediated signal amplification for RPPA. The subsequent data analysis revealed a strong correlation between both data sets validating AMSA as robust and reproducible (Pearson r = 0.89, p < 2.2^-16^) (Figure 3c).

### Comparison of AMSA to NIR-standard detection for the detection of low abundance proteins

**Figure 4:**
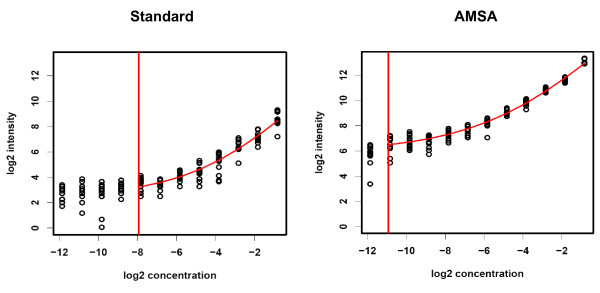
**Comparing the sensitivity of standard NIR detection and AMSA for JNK spike-in**. Dilution series of JNK (0.27 fg - 0.56 pg JNK per spot) in a colon cancer cell line lysate. Each dilution step was printed in 12 replicate spots. Samples were analyzed using standard NIR detection and AMSA on two-pad slides in parallel. Slides were scanned at intensity 5 on the Odyssey instrument. Vertical red lines indicate the calculated detection limits.

To test the advantage of AMSA also for the detection of rare proteins, serial dilutions of recombinant JNK protein were printed to determine the limit of detection (LOD) for AMSA and for standard NIR. A mixture of three human colon cancer cell lines (HT29, SW480, RKO) was used as matrix to prepare a 12-step JNK dilution series. All samples were printed in twelve replicate spots on 2-pad nitrocellulose-coated glass slides to compare both detection methods on a single slide. A curve fitting model (Additional file 1) was employed to analyze the raw data indicating an 8-fold lower limit of detection for AMSA when compared to standard detection: 0.55 femtogram JNK were detected per spot compared to 4.4 fg JNK per spot for standard NIR (Figure 4). Moreover, a sensitive discrimination even between small target protein differences was possible due to the improved linear range of antibody-mediated signal amplification.

**Figure 5:**
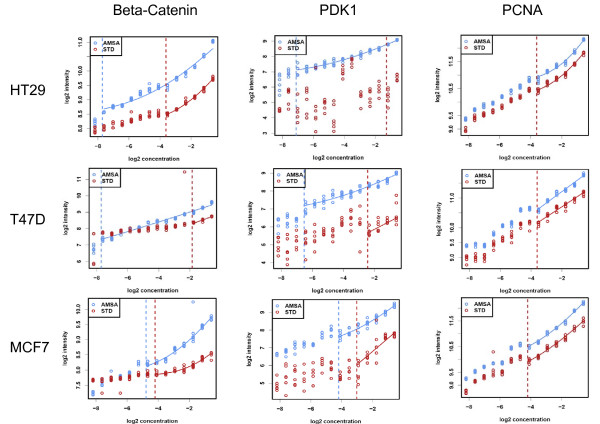
**Comparing the sensitivity of standard NIR detection and AMSA for endogenous proteins**. Detection of beta-Catenin, PDK1, and PCNA in the human colon cancer cell line HT-29 and in the human breast cancer cell lines T47D and MCF7 comparing AMSA with standard NIR. The limit of detection is indicated as red or blue line.

**Figure 6:**
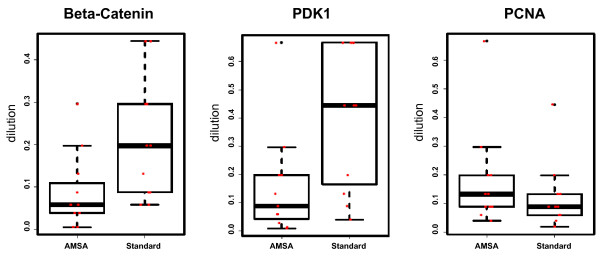
**Comparing detection limits (LOD) between standard NIR detection and AMSA **LOD for the detection of beta-Catenin, PDK1 and PCNA in eleven human cancer cell lines (SW480, HCT15, HCT116, SW620, HT29, T47D, BT474, MDA, SKBR3, HCC1954, MCF7) comparing AMSA with standard detection. The LOD is represented by the relative dilution step.

The general benefit of AMSA for the quantification of low abundance proteins was evaluated further using a set of eleven cancer cell lines. Cell line lysate samples were printed as 14-step serial dilutions and in two subarrays per slide. The first subarray was detected using standard NIR, the other with AMSA. The slides were scanned at different scanner sensitivity settings reflecting the fact that the AMSA detection generally produces stronger signals than the standard NIR detection. Thus, subarray images produced with scanner settings optimal for the AMSA readout, the corresponding subarray of the standard NIR detection was too dim to allow a reasonable comparison between both methods. For this reason, each slide was scanned at different scanner settings and for the direct comparison between standard NIR and AMSA images with comparable signal intensities were chosen (Figure 5). Results indicated that the detection of low abundant proteins indeed benefited from AMSA, as shown for the detection of beta-Catenin and PDK1. The limit of detection increased up to ten dilution steps for beta-Catenin, and PDK1 (Figure 5). However, when calculating the average LOD gain for all eleven cell lines a mean LOD gain of 4- and 5-fold was observed for beta-Catenin, and PDK1, respectively (Figure 6). The detection of abundant proteins such as PCNA did not benefit from AMSA (Figure 5 and 6). Thus, AMSA improved especially the detection of proteins expressed at a low level by reducing the scattering of weak signals and improving signal-to-noise ratios. Antibody-mediated signal amplification strategies promise to be useful for the RPPA-based quantitative analysis of systems-biology type of experiments as well as for the analysis of clinical samples.

## Discussion

Reverse phase protein microarray techniques allow for a highly parallel and quantitative analysis of signal transduction processes requiring only minute amounts of sample material. However, the protein of interest is frequently expressed at a low level and its detection requires highly sensitive methods. For this reason, a new NIR fluorescence-based amplification approach was developed [8,9]. To our knowledge, this is the first description of an amplification system using successive incubation steps with two species-specific secondary antibodies on protein microarrays.

Analyzing the expression level of 14 different proteins with moderate to high abundance in seven different human cancer cell lines by RPPA revealed that AMSA results corresponded to those obtained with standard NIR detection. Thus, the high correlation coefficient indicated that the signal intensity gain mediated by AMSA is reproducible. Furthermore, side-effects such as background signal intensity gain were analyzed with respect to the number of amplification cycles. Signal-to-noise ratios improved considerably after three amplification cycles, and four rounds of amplification were found to deliver the best result. Thus, after four amplification cycles the target specific signal intensity gain was higher than the increase of the local background. Moreover, a direct comparison between AMSA and standard NIR confirmed a lower detection limit for a specific protein spiked into a cell lysate as well as for endogenous proteins expressed at a low level.

Different types of signal detection strategies were reported for protein microarray applications. Colorimetric, chemiluminescence, fluorescence detection techniques, linear waveguide technologies [22], and rolling circle amplification [23] were so far successfully adapted to protein microarrays. Tyramide signal amplification is one of the most frequently used techniques to increase the sensitivity of reverse phase arrays. TSA is based on the catalyzed deposition of tyramide in close proximity of the secondary HRP-linked antibody [24,25]. The high velocity of enzymatic processes was suspected to cause signal saturation effects [26] and observed as sigmoidal curves in serial dilutions limiting the signal detection range on RPPA [7,27]. However, time-resolved measurements involving systems biology applications benefit from a linear correlation between sample concentration and signal intensity. Besides that, insufficient specificity has been reported for tyramide signal amplification strategies [19,28]. Ambroz et al. suggested that identical detection chemistries should be used for the RPPA-based analysis and for Western blot based antibody validation measures to reduce the risk of experimental artifacts [17]. Western blot detection indicated that indeed the use of a biotin/avidin-based signal amplification strategy produces unspecific bands presumably due to insufficient blocking of endogenous biotin-modified proteins. Biotin-modifications were identified on histones and presumably play a role in epigenetic mechanisms [29]. However, the use of AMSA circumvents the optimization of biotin-blocking procedures since no unspecific background signals were observed.

## Conclusions

We have shown that antibody-mediated signal amplification is a powerful tool for the specific and sensitive detection of signals on reverse phase protein microarrays over a large linear range. AMSA is easy to implement and could in principle be also adapted to other immunoassay formats. In addition, assay costs are low compared to commercial amplification strategies since only low concentrations of secondary antibodies are required as reagents.

## Abbreviations

AMSA: antibody-mediated signal amplification; HRP: horseradish peroxidase; IPAQ: infrared-based protein arrays with quantitative readout; NIR: near-infrared range; RPPA: reverse phase protein array; TSA: tyramide signal amplification

## Competing interests

The authors declare that they have no competing interests.

## Authors' contributions

JCB had the idea for the methodology; JCB, HM and UK planned the experiments; HM and JCB carried out the experiments; HF developed the curve fitting model and performed statistical analysis; SG performed statistical analysis; CS was responsible for the spotting runs; TB supervised the data analysis; TS provided clinical samples; UK and HS were responsible for the organization and course of the project; UK, HS, and SW were responsible for the equipment and recourses; JCB and UK wrote the manuscript; UK, JCB, HS, TB, SW, HM critically reviewed the content of the manuscript before submission. All authors read and approved the final manuscript.

## Supplementary Material

Additional file 1 **Species specificity of amplification antibodies RPPA-based analysis of human immunoglobulin and JNK dilution series (23 fg - 0.75 pg).** Serial dilutions were incubated with (a) no target specific detection antibody (b) anti human immunoglobulin (c) JNK specific detection antibody. 12 replicate spots were printed for each dilution step. Click here for file
