# Supplementary material for: Increasing the sensitivity of reverse phase protein arrays by antibody-mediated signal amplification
Source: Proteome Sci. 2010 Jun 22;8:36. doi: 10.1186/1477-5956-8-36 (PMC2908584; doi:10.1186/1477-5956-8-36)
Supplement: Additional file 1 — Species specificity of amplification antibodies RPPA-based analysis of human immunoglobulin and JNK dilution series (23 fg - 0.75 pg). Serial dilutions were incubated with (a) no target specific detection antibody (b) anti human immunoglobulin (c) JNK specific detection antibody. 12 replicate spots were printed for each dilution step. [file 1477-5956-8-36-S1.DOC]

**Supplemental Information - Data analysis**

In order to account for the broad range of signal intensities resulting from RPPA-based measurements, fluorescence readings were log2-transformed before performing further analysis. The signals were corrected for background effects using the “normexp” method from the limma package [1].

**Dilution Curve Fitting**

Serial dilutions are generally best described by fitting a sigmoid function [2]:


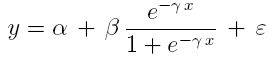
In this function y corresponds to the log2-transformed signal intensity, x to the log2-transformed concentration, ε the experimentally determined noise and α, β, γ are parameters used to fit the function. All three parameters were estimated from measurements based on the calculation of the detection limit by using a nonlinear median quantile regression via the R-package “quantreg” [3]).

**Data normalization**

After estimating the detection limit and parameters of the sigmoid function signal intensity readings were re-calculated for all steps of a dilution series using the functions described under curve fitting. The signal intensity corresponding to the highest concentration turned out to be a robust and reasonable measure reflecting the concentration of the protein of interest in a certain sample. Whenever the number of data points was too small to allow fitting of a sigmoid function - mostly due to low abundance of a protein or phosphoprotein - the median signal of the highest concentration was used for further analysis.

The final normalization was performed as described before: Signal intensity readings corresponding to the highest concentration were divided by the signal intensity from the corresponding spot of the FCF slide [4].

**Statistical comparison of different amplification methods**

Correlation analyses between two different detection methods were performed based on the Pearson correlation coefficient.

**Supplemental Figure 1**

Figure illustrates controls performed to set up the AMSA technology.

**Supplemental references**

[1] Smyth, G. K., Limma: linear models for microarray data. In:

'Bioinformatics and Computational Biology Solutions using R and

Bioconductor'. R. Gentleman, V. Carey, S. Dudoit, R. Irizarry, W.

Huber (eds), Springer, New York 2005, pages 397--420

[2] Hu, J., He, X., Baggerly, K. A., Coombes, K. R.*, et al.*, Non-parametric quantification of protein lysate arrays. *Bioinformatics* 2007, *23*, 1986-1994.

[3] Koenker, R., quantreg: Quantile Regression. *R package* 2008.

[4] Loebke, C., Sueltmann, H., Schmidt, C., Henjes, F.*, et al.*, Infrared-based protein detection arrays for quantitative proteomics. *Proteomics* 2007, *7*, 558-564.
